# Quality of life and quality of sleep in health professionals working in critical areas

**DOI:** 10.47626/1679-4435-2023-1190

**Published:** 2024-11-14

**Authors:** Hugo Machado Sanchez, Andre Luiz Sbroggio Junior, Eliane Gouveia de Morais Sanchez, Juciele Faria Silva, Luiz Almeida da Silva, Christiane Ricaldoni Givigiez, Ludmila Grego Maia

**Affiliations:** 1 Fisioterapia, Universidade Federal de Jataí, Jataí, GO, Brazil; 2 Medicina, Universidade de Rio Verde, Rio Verde, GO, Brazil; 3 Enfermagem, Universidade Federal de Catalão, Catalão, GO, Brazil; 4 Medicina, Universidade Federal de Jataí, Jataí, GO, Brazil; 5 Enfermagem, Universidade Federal de Jataí, Jataí, GO, Brazil

**Keywords:** quality of life, sleep, health personnel, work, qualidade de vida, sono, pessoal de saúde, trabalho

## Abstract

**Introduction:**

Emergency rooms and intensive care units in hospitals are physically and mentally stressful for health personnel and can negatively affect them.

**Objectives:**

To evaluate and to compare the quality of life and quality of sleep of emergency room and intensive care unit personnel.

**Methods:**

This is an analytical cross-sectional study conducted with 117 civil servants, including physicians, nurses, and licensed practical nurses. Sociodemographic questionnaires, the World Health Organization Quality of Life instrument-Abbreviated version, the Pittsburgh Quality Sleep Index and the Epworth Sleepiness Scale questionnaire were used.

**Results:**

The quality of sleep in women was worse than in men. Physicians scored lower in the psychological domain of quality of life than nurses. When the group of physicians was stratified between clinicians and surgeons, clinicians scored better in the physical domain and worse in the social relations and overall quality of life domains.

**Conclusions:**

The results showed that the psychological domain in nurses was less impaired than in physicians. Women also had poorer quality of sleep and were more likely to suffer from sleep disorders than men.

## Abstract (pt)

**Introdução:**

Os setores de emergência/urgência e terapia intensiva no ambiente hospitalar são locais de grande desgaste físico e mental aos profissionais atuantes nessas áreas, podendo gerar diversas repercussões nesses profissionais.

**Objetivos:**

Avaliar e comparar a qualidade de vida e a qualidade do sono dos profissionais que atuam nos setores de emergência/urgência e terapia intensiva.

**Métodos:**

Trata-se de um estudo analítico de corte transversal realizado com 117 profissionais do serviço público, sendo eles médicos, enfermeiros e técnicos de enfermagem. Os instrumentos utilizados foram o questionário sociodemográfico, o World Health Organization Quality of Life instrument-Abbreviated version, o Pittsburgh Quality Sleep Index e o questionário de Escala de Sonolência de Epworth.

**Resultados:**

A qualidade do sono das mulheres avaliadas apresentou-se pior que a dos homens. Os médicos obtiveram o menor escore no domínio psicológico da qualidade de vida quando comparados aos enfermeiros. Quando se estratificou o grupo de médicos entre clínicos e cirurgiões, verificou-se que os clínicos possuem melhor avaliação do domínio físico e pior avaliação dos domínios relações sociais e índice geral da qualidade de vida.

**Conclusões:**

De acordo com os resultados obtidos, houve um menor comprometimento dos profissionais enfermeiros em relação aos médicos no domínio psicológico. Ademais, o sexo feminino obteve qualidade do sono inferior e maior chance de distúrbios do sono quando comparado ao sexo masculino.

## INTRODUCTION

The World Health Organization (WHO) and the World Health Organization Quality of Life (WHOQOL) define quality of life (QoL) as individuals’ perceptions of their position in life in the context of the culture and value systems in which they live and in relation to their goals, expectations, standards and concerns.^1^

Specifically in health care, improving QoL is considered to be an outcome to be achieved after care is provided, also in public policies for health promotion and disease prevention. Therefore, information on QoL has been used as an indicator to assess the effectiveness and impact of certain treatment or prevention programs on groups/individuals, whether they are ill or are likely to become ill, and professionals in the field are using the term more frequently.^1,2^

The medical career has admittedly stressful aspects, such as the need to dedicate a lot of time, involving a lot of personal responsibility, continuous exposure to the suffering of patients and their families, high workloads (WLs), and multiple jobs. As a result, more than half of physicians have psychiatric complaints such as anxiety and depression, not to mention severe fatigue, and statistics show that 5% of them feel hopeless, unhappy, and suicidal.^3^

Health personnel are required to be agile and precise to make good decisions, which can sometimes be life-threatening. It is therefore important that they have a good QoL and quality of sleep. High WLs, particularly at night shifts, can have an unsatisfactory effect on the recovery of patients in intensive care units (ICUs) and emergency rooms, since sleep deprivation favors inattention. Some authors attribute the poor QoL of health personnel to their contact with weakened patients; however, the fact that resting the body and mind effectively results in more competent treatment should not be overlooked.^4,5^

A study including nursing assistants, licensed practical nurses, and nurses showed that their routine and WL are perceived as intense, to the point of having an effect on their private and personal lives.^6^ Occupational stress can negatively affect their performance, leading to reduced productivity, as well as their levels of satisfaction and QoL.^7^

Hospital work experience, especially in highly complex settings such as ICUs and emergency rooms, has special characteristics such as night shifts, rotating shifts, overtime, and on-call shifts. These characteristics can cause physical and mental strain on workers, and circadian and homeostatic differences can influence their preferences, which vary according to their adaptability to the working routine. However, when workers and their endogenous rhythms are not prioritized, their shifts are often determined according to management requirements, regardless of their circadian cycles. This leads to a lower capacity to perform tasks, especially those associated with cognition, which negatively affects the overall QoL of health personnel.^8^

Psychological factors, sleeping conditions, and one’s lifestyle can affect the quality of sleep. In addition, sleep plays a decisive role in memory consolidation, which suggests that it facilitates processing new information. Thus, even acute sleep deprivation can negatively affect learning, as it impairs processing new information.^9^ Sleep disorders, such as sleepiness, characterized as a decreased ability to maintain wakefulness, an increased propensity to fall asleep, and the likelihood of falling asleep, can cause poor performance at school, at work, and in family and social relationships, and are associated with increased accident risks.^10,11^

The fact that physicians and nurses work in shifts causes them to experience distress not only because of their loss of actual sleeping hours, but also because these working conditions can affect other lifestyle factors, such as food intake, level of physical activity and, consequently, metabolic patterns depending on the time of day. Workers who report irregular work shifts are more susceptible to errors and chronic fatigue associated with these response variations. It is also worth remembering that excessive daytime sleepiness (EDS) is an important public health and clinical problem, affecting 10%-25% of the population.^12^

It is therefore important to realize that the deterioration of nocturnal performance can be affected both by acute and chronic sleep deprivation and by the disruption of biological rhythms, especially when work is performed in the opposite time of the day. Several studies have shown that mental performance at night is comparable to performance after alcohol intake or a day following sleep deprivation.^13^

Poor sleep has a negative and direct influence on QoL, as people who have poor sleep generally have poor relationships. Thus, sleep deprivation leads to social isolation, anxiety, and high stress, factors that impact on social relationships. In addition, those who have poor or no sleep during the night often try to compensate during the day in an attempt to have restful sleep, culminating in less social interaction and low self-esteem, which leads to an onset of physical symptoms such as headaches and back pain.^14^

This study aimed to evaluate and compare QoL and its domains among health personnel (physicians, nurses, and licensed practical nurses) working in emergency rooms and ICUs. The study also aimed to verify the correlation with the dependent variables (time since graduation [TG], WL, age, sex, chronic diseases, and physical activity), comparing these data with the results provided by the Epworth Sleepiness Scale (ESS) and the Pittsburgh Sleep Quality Index (PSQI).

## METHODS

This is a cross-sectional analytical study conducted in emergency rooms and ICUs of three hospitals, and in the Serviço de Atendimento Móvel de Urgência (SAMU, Mobile Emergency Care Service), in a southwestern municipality in Goiás, Brazil.

This study was approved by the Research Ethics Committee, complying with the ethical standards of research involving human beings, report No. 042821/2014.

A total of 117 volunteer health personnel participated in the study, drawn from a nonprobabilistic sample and with no refusal to participate. All the participants work in the SAMU, emergency rooms, or ICUs, including 39 physicians, 36 nurses, and 42 licensed practical nurses. These health personnel are based in the emergency rooms and ICUs and, once informed about the study, they signed an informed consent form. All the workers worked either for SAMU or for a medium-sized public hospital. Participants were sampled on a nonprobabilistic, convenience basis, and were invited individually to participate in the study.

The study included physicians, nurses, and licensed practical nurses working in emergency rooms, ICUs, and SAMUs, regardless of their sex, ethnicity, or age group. All were registered with their professional regulatory boards.

The study excluded people with physical disabilities, due to impairment of the physical domain; health personnel who were on vacation for more than 15 days or for any other reason that prevented them from working; pregnant women who were still working; those receiving psychiatric treatment; and people with obstructive sleep apnea (self-reported).

Data were collected simultaneously at all the sites, using four instruments: the first refers to sociodemographic characteristics; the second is a generic instrument proposed by the WHO to assess QoL, called the WHOQOL-Bref; the third is the PSQI, a qualitative and quantitative questionnaire on the quality of sleep in the previous month; and finally, the ESS, a self-reporting questionnaire which, as it is considered easy to understand, quick, and simple to fill in, can be used in clinical routine both for diagnostic purposes and monitoring the response to prescribed treatments, both in epidemiological studies and in clinical research. All data collection occurred in person, through interviews conducted by trained investigators, ensuring the confidentiality of participants according to the Comissão Nacional de Ética em Pesquisa (CONEP, Brazilian National Research Ethics Commission) Resolution No. 466 of 2012.

Sociodemographic characteristics were collected using a questionnaire designed by the authors of this study. This questionnaire was sent to three experts in the field for suggestions and corrections, before being applied, and was then subjected to a pre-test, which also resulted in corrections. It is a structured questionnaire, subdivided into personal data, work-related data, and health data.

The second questionnaire, the WHOQOL-Bref, is an abbreviated version of the WHOQOL-100, created by the WHO QoL Group in 1998 and validated in Portuguese by Fleck et al.^15^ This questionnaire showed good psychometric performance, is user-friendly, and has satisfactory internal consistency and reliability characteristics. It consists of 26 items distributed into four domains, an overall index, and four indices per domain, calculated using the means of the items, with scores from 0 to 100. The internal consistency, with α = 0.91, was calculated for the domains in this study. All the questionnaires were self-administered; however, in case of any questions, the investigators were available to help the respondents, making it a well-assisted application. The higher the scores, the better the QoL of the respondents.

The third questionnaire is the PSQI, developed in 1989 by Buysse et al.^16^ and translated, adapted, and validated into Brazilian Portuguese by Bertolazi et al.^5^ The PSQI assesses the quality of sleep over the previous month, which is an intermediate period compared to questionnaires that only assess the previous night, which are unable to detect dysfunction patterns, and those that assess the previous year, which are unable to indicate the severity of the problem at the moment. As a result, an important feature of this questionnaire is enabling a combination of quantitative and qualitative information, distinguishing between good and poor sleepers.

Finally, the fourth questionnaire is the ESS, published in 1991 by Johns.^17^ The scale was developed to assess excessive daytime sleepiness, referring to the possibility of dozing off in everyday situations. In 2009, Bertolazi et al.^5^ translated it into Brazilian Portuguese and validated it in Brazil. The ESS assesses the ability of individuals to fall asleep while performing any of the eight daily tasks described in the questionnaire, some of which are considered soporific. In this questionnaire, the respondent is considered to be suffering from excessive daytime sleepiness when their score exceeds 10, in a range from 0 to 24.^5^

The data were analyzed using SPSS^®^ 22.0. The Kolmogorov-Smirnov (KS) test was used to analyze the data distribution. Data distribution was compared using the chi-squared test. The means of the two groups with a normal distribution were compared using Student’s *t*-test for unrelated samples. To compare the means of more than two groups, one-way analysis of variance (ANOVA) with Tukey’s *post-hoc* test was used. The Pearson correlation coefficient was also used to analyze correlations between variables.

All tests were applied considering a significance level of 5% (α = 0.05).

## RESULTS

Categorical variables are presented as frequencies, with absolute numbers and percentages. Continuous quantitative variables are presented with their means and standard deviation (SD).

A total of 117 health personnel working in emergency departments completed the questionnaires, including 39 physicians, 36 nurses, and 42 licensed practical nurses. The sample consisted of 47 men, with a mean age of 38.90 ± 6.14 years, and 70 women, with a mean age of 35.5 ± 8.97 years. The overall mean age was 36.90 ± 9.9 years. As for their workplace, 96 worked in emergency rooms or the SAMU, and 21 in the ICU. The mean age of those working in emergency rooms or the SAMU was 35.93 ± 7.79 years, and 41 ± 4.87 years for those in ICUs.

Table 1 shows the frequency of sociodemographic and lifestyle variables of physicians, nurses, and licensed practical nurses working in emergency departments such as Unidade de Pronto Atendimento (UPA, Emergency Care Unit) and SAMU, and ICUs.

**Table 1:** Relative percentage frequency for the sociodemographic, occupational, and lifestyle variables of the volunteers (n = 117)

Characteristics	n	%
Sex	117	
Female	70	60.34
Male	47	39.66
Marital status		
Single	43	36.75
Married	62	52.99
Stable union	5	4.27
Divorced	7	5.98
Occupation		
Physicians	39	33.33
Nurses	36	31.00
Licensed practical nurses	41	35.36
Workplace		
SAMU	13	11.11
UPA	83	70.94
ICU	21	17.95
Shifts per week		
1	5	4.27
2	22	18.80
3	23	19.66
4	37	31.62
5	21	17.95
6	8	6.84
8	1	0.86
Weekly WL (hours)		
12	5	4.27
24	22	18.80
36	23	19.66
48	37	31.62
60	21	17.95
72	8	6.84
96	1	0.86
Specialization		
Yes	54	46.15
No	63	53.85
Type of specialization		
Clinical	39	33.33
Surgical	14	11.95
None	64	54.70
Alcohol use disorder		
Yes	41	35.04
No	76	64.96
Physical exercise		
Yes	55	47.01
No	62	52.99
Chronic disease(s)		
Yes	16	13.68
No	101	86.32

SAMU = Serviço de Atendimento Móvel de Urgência (Mobile Emergency Care Service); UPA = Unidade de Pronto Atendimento (Emergency Care Unit); UTI = intensive care unit; WL = workload.

A comparative analysis between marital status, QoL and its domains was performed. According to this analysis, being single was statistically lower (p = 0.009) than being married in the physical domain.

Table 2 shows the comparison between sex and the dependent variables.

**Table 2:** Comparison using Student’s *t*-test between the independent variable sex and the dependent variables (n =117)

Domain	Sex	Mean ± SD	p-value
Physical	Male	74.23 ± 15.37	0.206
	Female	71.67 ± 14.46	
Psychological	Male	69.44 ± 15.43	0.413
	Female	66.57 ± 15.68	
Social relations	Male	67.74 ± 18.88	0.470
	Female	65.54 ± 16.35	
Environmental	Male	63.08 ± 13.58	0.254
	Female	60.75 ± 13.40	
QoL	Male	68.44 ± 12.33	0.227
	Female	65.92 ± 11.35	
PSQI	Male	5.82 ± 3.19	0.024^*^
	Female	7.52 ± 4.08	
ESS	Male	9.38 ± 4.95	0.549
	Female	10.14 ± 5.60	

SD = standard deviation; ESS = Epworth Sleepiness Scale; PSQI = Pittsburgh Sleep Quality Index; QoL = quality of life.

* p < 0.05.

Table 3 shows the descriptive data and ANOVA and chi-square comparisons of the sociodemographic characteristics among occupations.

**Table 3:** Descriptive data and ANOVA and chi-square comparisons of the sociodemographic characteristics among occupations

	Physicians (n or mean)	Nurses (n or mean)	Licensed practical nurses (n or mean)	p-value
Sex				
Male	21	12	14	0.113
Female	18	24	28	
Marital status				
Single	12	14	17	0.223
Married	27	22	13	
Stable union	0	0	5	
Divorced	0	0	7	
Alcohol use disorder				
Yes	14	12	15	0.967
No	25	24	27	
Physical exercise				
Yes	29	16	10	0.059
No	10	20	32	
Chronic disease(s)				
Yes	10	4	2	0.074
No	29	32	40	
Length of education	10.28	8.03	8.79	0.089
Workload	38.77	43.06	45.52	0.072
Shifts	3.33	3.69	3.90	0.456

Table 4 shows a comparison test between the groups of health personnel (A × B) working in emergency rooms in relation to QoL and its domains.

**Table 4:** Comparison among the groups of health personnel working in emergency rooms in relation to QoL and its domains using one-way ANOVA and Tukey’s *post-hoc* test (n = 117)

Dependent variable Domain	Compared occupations	Compared occupations	p-value
Physical	Physicians	Nurses	0.94
		Licensed practical nurses	0.94
	Nurses	Physicians	0.94
		Licensed practical nurses	1.00
	Licensed practical nurses	Physicians	0.94
		Nurses	1.00
Psychological	Physicians	Nurses	0.00^*^
		Licensed practical nurses	0.13
	Nurses	Physicians	0.00^*^
		Licensed practical nurses	0.20
	Licensed practical nurses	Physicians	0.13
		Nurses	0.20
Social relations	Physicians	Nurses	0.85
		Licensed practical nurses	0.98
	Nurses	Physicians	0.85
		Licensed practical nurses	0.92
	Licensed practical nurses	Physicians	0.98
		Nurses	0.92
Environmental	Physicians	Nurses	0.67
		Licensed practical nurses	0.64
	Nurses	Physicians	0.67
		Licensed practical nurses	1.00
	Licensed practical nurses	Physicians	0.64
		Nurses	1.00
QoL	Physicians	Nurses	0.56
		Licensed practical nurses	0.75
	Nurses	Physicians	0.56
		Licensed practical nurses	0.93
	Licensed practical nurses	Physicians	0.75
		Nurses	0.93

ANOVA = analysis of variance; QoL = quality of life.

* p < 0.05.

Table 5 shows the comparison using Student’s *t*-test with the variable type of specialization (clinical or surgical).

**Table 5:** Comparison using Student’s *t*-test with the independent variable type of specialization (clinical or surgical)

Domain	Specialization	Mean ± SD	p-value
Physical	Clinical	73.70 ± 15.19	0.02^*^
	Surgical	71.84 ± 14.57	
Psychological	Clinical	67.05 ± 15.95	0.12
	Surgical	68.30 ± 15.36	
Social relations	Clinical	65.16 ± 9.62	0.04^*^
	Surgical	67.5 ± 15.25	
Environmental	Clinical	61.29 ± 13.53	0.20
	Surgical	62.03 ± 13.50	
QoL	Clinical	66.57 ± 12.49	0.02^*^
	Surgical	67.25 ± 11.21	
Quality of sleep	Clinical	0.90 ± 0.73	0.83
	Surgical	1.16 ± 0.83	
PSQI	Clinical	6.48 ± 3.51	0.48
	Surgical	7.17 ± 4.09	
ESS	Clinical	9.31 ± 5.66	0.75
	Surgical	10.28 ± 5.04	

SD = standard deviation; ESS = Epworth Sleepiness Scale; PSQI = Pittsburgh Sleep Quality Index; QoL = quality of life.

* p < 0.05.

A significant difference was found between those who drank alcohol and those who did not, at the 5% probability level, with the group who did not drink alcohol scoring higher on the PSQI (p = 0.04) than the group who drank alcohol. We compared QoL and its domains with the independent variables: physical exercise, whether or not they had a chronic disease and WL. No statistically significant results were found (p < 0.05).

The dependent variable TG, when categorized by occupation, showed that the TG of physicians was statistically higher than the TG of nurses (p = 0.027) and licensed practical nurses (p = 0.049).

Tables 6 and 7 show correlations using Pearson’s correlation coefficient. Table 6 shows the correlation between QoL and its domains, and between domains and QoL with age and TG, WL, shifts per week, PSQI and ESS total score. Table 7 shows the correlation between age and TG, WL, shifts per week, PSQI, and ESS total score.

**Table 6:** Correlation between domains and QoL with age and TG, WL, shifts per week, PSQI, and ESS total score using Pearson’s correlation coefficient (n = 117)

Domain	Age		TG		Weekly WL		Shifts per week		PSQI		ESS	
	r	p	r	p	r	p	r	p	r	p	r	p
Physical	0.17	0.55	0.15	0.98	-0.11	1.00	0.28	0.76	-0.11	0.83	-0.03	0.57
Psychological	0.55	0.57	0.31	0.73	0.12	0.26	0.16	0.79	-0.28	0.23	-0.18	0.04^*^
Social relationships	0.75	0.42	0.80	0.39	0.10	0.03^*^	0.24	0.01^*^	-0.98	0.76	-0.14	0.11
Environmental	0.20	0.82	0.15	0.87	0.89	0.38	0.12	0.18	0.86	0.29	0.10	0.04^*^
QoL	0.10	0.53	0.10	0.28	0.11	0.23	0.10	0.04^*^	-0.53	0.35	0.10	0.26

WL = workload; ESS = Epworth Sleepiness Scale; PSQI = Pittsburgh Sleep Quality Index; QoL = quality of life; TG = time since graduation.

* p < 0.05.

**Table 7:** Correlation between age and TG, WL, shifts per week, PSQI, and ESS total score using Spearman’s correlation coefficient (n = 117)

	Age		TG		WL		Shifts per week		PSQI		ESS	
	r	p	r	p	r	p	r	p	r	p	r	p
Age	1.00	0.00	0.72^*^	0.00	0.15	0.26	0.13	0.15	0.97	0.30	-0.03	0.97
TG	0.72	0.00^*^	1.00	0.00	0.98	0.00	0.11	0.23	0.27	0.77	0.09	0.92
WL	0.10	0.26	0.98	0.00^*^	1.00	0.00	0.02	0.01	-0.02	0.83	-0.01	0.85
Shifts per week	0.13	0.15	0.11	0.23	0.90	0.00^*^	1.00	0.00	0.01	0.908	-0.04	0.66
PSQI	0.20	0.82	0.15	0.87	0.89	0.38	0.12	0.18	0.86	0.29	0.30	0.01^*^
ESS	0.12	0.19	0.09	0.29	0.15	0.86	-0.40	0.61	0.33	0.00^*^	1.00	0.00

WL = workload; ESS = Epworth Sleepiness Scale; PSQI = Pittsburgh Sleep Quality Index; TG = time since graduation.

* p < 0.05.

Chi-square tests were used to link sex and PSQI, which showed a negative association between women (p = 0.02) and men. Associations were also found among ESS (p = 0.67), occupations, and PSQI (p = 0.61), both of which were not statistically significant.

## DISCUSSION

Since the 1990s, QoL has been a hot topic on the scientific scene, especially in populations with specific diseases and their families. However, when QoL and its domains are addressed to health personnel, investigations have declined sharply.^18^

This study found that single marital status was statistically lower in the physical domain than married, as in Webster et al.,^19^ a study with workers subjected to similar stressful conditions. Single individuals were statistically lower in all domains, while married ones were less predisposed to depression and burnout. This was due to the presence of emotional relationships and support, which help to reduce the feeling of discomfort and fatigue, which is one of the aspects of the physical domain.

The comparison between groups of occupations revealed that only the psychological domain showed a statistical difference when comparing physicians with nurses. Paschoa et al.^20^ found a negative correlation between the psychological domain and nurses working in ICUs, which was justified because nurses have more personal contact with patients and spend a large part of their shift caring for them. No significant differences were found in the environmental, physical, social relations, and perceived QoL domains between the groups of health personnel, since these groups have their own peculiarities and roles within the same workplace, although subject to the same stressors and shift patterns, consistent with other reports in the literature.^21^

When compared to the surgical specialty, the clinical also obtained higher scores in the physical domain. This domain involves questions about satisfaction in relation to pain, medical treatment, sleep, energy for everyday life, work, and chores/errands.^22^ In this sense, it can be inferred that physicians working in the clinical setting are less attached to rules and routine, which ultimately creates greater opportunities to engage in activities that improve QoL and their overall domains.^23^ Unlike the results found by Bohrer et al.,^24^ who assessed surgeons and clinicians working in Germany and found a reduced QoL among surgeons, this study found a higher QoL, especially in the social relationships domain. This difference may be explained by the different working conditions found here.

As for sleep, women had a statistically higher PSQI than men, which indicates poorer sleep quality. This finding agrees with the study by Spitz et al.,^25^ who compared quality of sleep and QoL in academics in their freshman year at university, a stressful factor equivalent to that experienced by the professionals in this study, using the same questionnaire applied in this study and obtaining a similar result. This can be explained by the “architecture” of sleep in women, which varies during their menstrual cycle, pregnancy, and menopause, affecting the quality of sleep due to changing in hormone levels experienced in the body.

Women are more likely to suffer from sleep disorders, since their sleep tends to be more fragmented and less continuous than that of men. In addition, the stressful social context in which women are placed (social demands related to work, family care, and esthetical aspects) can lead to unhealthy behaviors that negatively affect sleep. This results in significantly increased sleep latency and decreased sleep efficiency and quality.^26^

External issues, such as marital relationships, household, professional, and financial responsibilities, can have a more significant influence on the quality and quantity of sleep in women than in men. These factors can even cause dysfunctions in the sleep-wake rhythm. In addition, stressful situations can release cortisol into the bloodstream, a hormone that affects sleep quality and can cause anxiety and symptoms of depression, even to a mild degree. These effects contribute to increased sleep latency and an overall negative impact on sleep quality.^27^

The group of alcohol users had a slight advantage in the PSQI. Alcohol is probably the most widely used sleep-inducing substance. However, the repercussions of alcohol intake on sleep can lead to different results. In the first 3 hours after drinking alcohol, a decrease in sleep latency and rapid eye movement (REM) sleep occurs, as well as an increase in the non-REM phase (NREM). In the second half of rest, sleep can be interrupted by symptoms such as gastric irritation, headache, nightmares, tachycardia, and sweating. In addition, between 36% and 72% of alcohol users still suffer from insomnia after months of abstinence.^28,29^ However, this study only assessed drinking habits and not drinking frequency. A limitation of the PSQI is it is restricted to the previous 3 months, which can create a misleading perception of quality of sleep among alcohol users, which may represent a false impression of improvement.^30^

The Pearson correlation test results are consistent with the findings in the literature. Scheffer et al.^31^ reported that a WL of 40 to 60 hours per week is the most prevalent in all age groups. This WL is 39.5% prevalent among the youngest, 36.7% among the oldest, and almost half (47.5%) among middle-aged health personnel, which is not very different from the correlation between WL and TG and shifts and WL.^28,29^

As for the correlations with the QoL domains, positive correlations were found between the physical domain and the ESS total score. This is consistent with Costa,^32^ a study conducted with night shift workers, which found that the physical domain is directly related to sleep. The main items in this domain focus on the presence of pain or discomfort, dependence on medication, satisfaction with sleep, ability to work, and daily activities, for example.

It is worth noting that quality of sleep may have influenced the mean value, since most of the emergency personnel work night shifts. This group has an imbalance between meal times and food quality (frozen, pre-cooked food) and an increased intake of caffeinated drinks. This can lead to digestion problems, heartburn, constipation, a propensity to cardiovascular problems such as high blood pressure, ischemic diseases, increased risk of heart attacks, and angina pectoris, for instance. This condition can make people persistently sleepy, which can affect their ability to work, their energy levels, and their capacity to satisfactorily perform their daily tasks.^33^

In addition to these factors, the correlation between the physical and environmental domains can be explained because the environmental domain asks questions related to safety and leisure. As most health personnel do not have access to recreation and leisure due to fatigue after strenuous working hours, this influences the perception of the environmental and physical scores.^33^

Positive correlation was also found between the social relations domain and the WL and number of shifts per week. This can be explained because social interaction has a direct influence on the results. Health personnel who spend more time with the multidisciplinary emergency team tend to mature their social interaction, resulting in a more positive perception of this domain.

The results of this study deserve special attention due to the importance of work in the field of health prevention for the population studied and the scarcity of similar studies in the southwestern region of Goiás, Brazil. As it is common in all cross-sectional studies, this study alone cannot determine QoL and quality of sleep over the years. Thus, a longitudinal study is required.

Although these occupations require a great deal of knowledge, many specific characteristics are found in each of those assessed. This study did not assess psychological and emotional aspects, working in the Sistema Único de Saúde (SUS, Brazil Unified Health System) or in private hospitals exclusively, and compensation. Even though not all current health personnel were assessed, emergency rooms and ICUs are known to be stressful settings and cause occupational changes in the QoL and sleep.

## CONCLUSIONS

The results showed that nurses were more psychologically impaired than physicians. Those with a clinical specialization had higher scores in the physical domain, while those with a surgical specialization had better scores in the social relations and perceived QoL domains. Women had the worst quality of sleep scores and were more likely to have sleep disorders than men. On the other hand, the group of alcohol users had the best quality of sleep scores.

This study could not determine the relationship between QoL and sleep over their length of service. These findings are an essential step for the scientific community to contribute to making sound decisions, with a view to facilitating the management of aspects that compromise QoL and sleep. These data can provide a basis for developing strategies aimed not only at QoL at work, but also at interventions, focusing on preventive approaches in situations that negatively affect the health of health personnel in these critical departments.
